# Perforating Arteries of the Lemniscal Trigone: A Microsurgical Neuroanatomic Description

**DOI:** 10.3389/fnana.2021.675313

**Published:** 2021-08-26

**Authors:** Santino Ottavio Tomasi, Giuseppe Emmanuele Umana, Gianluca Scalia, Roberto Luis Rubio-Rodriguez, Giuseppe Raudino, Julian Rechberger, Philipp Geiger, Bipin Chaurasia, Kaan Yaǧmurlu, Michael T. Lawton, Peter A. Winkler

**Affiliations:** ^1^Department of Neurological Surgery - Christian Doppler Klinik, Salzburg, Austria; ^2^Department of Neurosurgery, Paracelsus Medical University Salzburg, Salzburg, Austria; ^3^Laboratory for Microsurgical Neuroanatomy - Christian Doppler Klinik, Salzburg, Austria; ^4^Department of Neurosurgery, Cannizzaro Hospital, Trauma Center, Gamma Knife Center, Catania, Italy; ^5^Neurosurgery Unit, Highly Specialized Hospital and of National Importance “Garibaldi”, Catania, Italy; ^6^Skull Base and Cerebrovascular Laboratory, University of California, San Francisco, San Francisco, CA, United States; ^7^Department of Neurological Surgery, University of California, San Francisco, San Francisco, CA, United States; ^8^Department of Otolaryngology - Head and Neck Surgery, University of California, San Francisco, San Francisco, CA, United States; ^9^Department of Neurosurgery - Humanitas, Istituto Clinico Catanese, Catania, Italy; ^10^Department of Neurosurgery, Neurosurgery Clinic, Birgunj, Nepal; ^11^Department of Neurosurgery, University of Virginia, Charlottesville, VA, United States; ^12^Department of Neurosurgery, Barrow Neurological Institute, St. Joseph's Hospital and Medical Center, Phoenix, AZ, United States

**Keywords:** lemniscal trigone, dorsolateral midbrain perforating zone, microsurgical anatomy, arterial capillary network, perforating arteries, anatomical variability

## Abstract

**Background:** The perforating arteries in the dorsolateral zone of the midbrain play a crucial role in the functions of the brain stem. Their damage due to herniation, pathological lesions, or surgery, favored by the narrow tentorial incisura, can lead to hemorrhages or ischemia and subsequently to severe consequences for the patient.

**Objective:** In literature, not much attention has been directed to the perforating arteries in the lemniscus; in fact, no reports on the perforators of this anatomical region are available. The present study aims to a detailed analysis of the microanatomy and the clinical implications of these perforators, in relation to the parent vessels. We focused on the small vessels that penetrate the midbrain's dorsolateral surface, known as lemniscal trigone, to understand better their microanatomy and their functional importance in the clinical practice during the microsurgical approach to this area.

**Methods:** Eighty-seven alcohol-fixed cadaveric hemispheres (44 brains) without any pathological lesions provided the material for studying the perforating vessels and their origin around the dorsolateral midbrain using an operating microscope (OPMI 1 FC, Zeiss). Measurements of the perforators' distances, in relation to the parent vessels, were taken using a digital caliper.

**Results:** An origin from the SCA could be found in 70.11% (61) and from the PCA in 27.58% (24) of the hemispheres. In one hemisphere, an origin from the posterior choroidal artery was found (4.54%). No perforating branches were discovered in 8.04% of specimens (7).

**Conclusion:** The perforating arteries of the lemniscal trigone stem not only from the superior cerebellar artery (SCA), as described in the few studies available in literature, but also from the posterior cerebral artery (PCA). Therefore, special attention should be paid during surgery to spare those vessels and associated perforators. A comprehensive understanding of the lemniscal trigone's perforating arteries is vital to avoid infarction of the brainstem when treating midbrain tumors or vascular malformations.

## Introduction

Although in the literature we can find some studies about the lemniscal trigone (Alezais and D'Astros, 1892; Testut and Latarjet, 1949; Duvernoy, 1978; Ardeshiri et al., 2006, 2007)—the region limited by the lateral mesencephalic sulcus, the superior cerebellar peduncle, and the inferior colliculus and its brachium—a precise microanatomical description of this area's microvascular network is still lacking, especially regarding the origin, number, and individual variability of the perforating arteries.

This study aims to provide a microsurgical anatomical definition of the perforating vessels and to name their parent vessels, which stem from the dorsolateral midbrain perforation zone's arterial capillary network. Furthermore, we also aim to define this capillary network's origin and course in the lemniscal trigone, by a thorough anatomical dissection, to clarify the microsurgical implications in the clinical practice and provide better microsurgical approaches to the brain stem.

### Anatomical Landmarks

The mesencephalon consists of the cerebral peduncles, the tegmentum, and the tectum. The upper border is formed by the sulcus between the optic tracts and the cerebral peduncles. Inferiorly, the mesencephalon is demarcated from the pons by the pontomesencephalic sulcus. The ventral aspect is represented by the cerebral peduncles and the posterior aspect by the quadrigeminal plate. The lateral mesencephalic sulcus separates the cerebral peduncles from the tegmentum.

### Lateral Mesencephalic Sulcus (LMS)

The lateral mesencephalic sulcus is located at the level of the lateral mesencephalic vein. It runs between the peduncular and tegmental surfaces of the midbrain, overlooking the middle incisural space (Ono et al., 1984), and caudally, in a concave trajectory, from the medial geniculate body to the pontomesencephalic sulcus. The posterior P2 segment (P2P) superiorly, the medial posterior choroidal artery centrally, and the cerebellomesencephalic segments of the SCA, trochlear nerve, and tentorial edge inferiorly all pass across the LMS (Cavalcanti et al., 2016). The safe entry zone is located between the substantia nigra anterolaterally and the medial lemniscus posteriorly. The dissection is limited anterolaterally by the oculomotor nerve fibers that cross from the red nucleus to the substantia nigra. In literature it has been reported that the average total length of the sulcus ranges from 7.4 to 13.3 mm, with a mean surgical corridor length of 8.0 mm, ranging from 4.9 to 11.7 mm (Recalde et al., 2008).

The acoustic fasciculus, a part of the auditory system, runs in this region, where the inferior colliculus plays the role of relay point for the auditory pathway, as it conducts information from the inner ear to the auditory cortex (Snell, 1999; Naidich et al., 2009; Driscoll and Tadi, 2020).

Although the inferior colliculus also receives inputs from non-auditory sources, it is primarily involved in auditory functions, since its efferent fibers project mostly to the thalamus's medial geniculate nucleus (Gruters and Groh, 2012; Ruchalski and Hathout, 2012; Mei et al., 2013).

As most of the midbrain tracts and nuclear groups are located at the lemniscal trigone level, derangements in this small area and its vascular perforating supply may have widespread effects, resulting in several neurological deficits. Primarily, lesions of the lemniscal trigone (e.g., tumors, hemorrhages, and other injuries) could cause auditory problems, such as cortical deafness, tinnitus, hyperacusis, audiogenic seizures, auditory agnosia, deficits in contralateral hemispace sound localization, and difficulty recognizing speech among other concurrent noises on the ipsilateral side (Litovsky et al., 2002; Stimmer et al., 2009; Niu et al., 2013; Poliva et al., 2015; Párraga et al., 2016; Kwee et al., 2017).

If the damage extends beyond the trigone's boundaries, other deficits could appear, such as a contralateral hemisensory loss (with involvement of the spinothalamic tract) and/or vertical diplopia (if the trochlear nerve is injured) (Driscoll and Tadi, 2020).

Therefore, auditory evoked potentials could be useful for diagnosing pathologies in the auditory system and monitoring intraoperatively the auditory pathways during surgery in this area.

## Methods

Eighty-seven alcohol-fixed cadaveric hemispheres from 44 brains without any pathological lesions (one hemisphere was damaged and therefore discarded) provided the material for studying the perforating vessels and their origin around the dorsolateral midbrain, using an operating microscope (OPMI 1 FC, Zeiss, Oberkochen, Germany).

The brains used in the study came from the Institute of Pathological Anatomy of Munich, Germany, and, after our study, they were used for the annual microsurgical neuroanatomy course in our research laboratory. Ethical review and approval and written informed consent were not required for the study on human participants, in accordance with the local legislation and institutional requirements. Written informed consent was not obtained from the individual(s) for the publication of any potentially identifiable images or data included in this article.

We used a digital camera (Nikon D1, Tokyo, Japan) for micro-photographs of relevant structures. Measurements of the distances between the inferior colliculus and inferior lateral sulcus, the inferior lateral sulcus and superior lateral sulcus, and the inferior colliculus and superior lateral sulcus were taken using a digital caliper (Digimatic CD-15B, Mitutoyo, Kawasaki, Japan).

The study included 24 men (54.54%) and 20 women (45.46%), with a mean age of 34.3 years (range 20–55 years).

First, the brain stem was observed from a lateral view, after gentle retraction of the temporal lobe and cerebellum, to identify, following the basilar artery, the superior cerebellar artery (SCA) inferiorly, the posterior cerebral artery (PCA) superiorly, and the trochlear nerve, then the lateral mesencephalic sulcus and the lateral mesencephalic vein. The PCA and the SCA with its branches, the medial superior cerebellar artery (MSCA) and lateral superior cerebellar artery (LSCA), were carefully dissected to find, if present, the penetrating branches in the lemniscal trigone and their origin.

## Results

The penetrating branches originated from the PCA (P2 segment) in 24 (27.58%) of the 87 hemispheres and from the SCA (ambient segment) in 61 (70.11%) hemispheres. In 2 (2.29%) hemispheres, they originated directly from a small branch of the basilar artery (BA) (Table 1).

**Table 1:** Origin of perforators from the PCA, SCA and BA (with different combinations).

**Perforators**	**Hemispheres *n =* 87 (100%)**
PCA	24 (27.58%)
SCA	61 (70.11%)
BA	2 (2.29%)

### Origin From Posterior Cerebral Artery

In a more detailed analysis of the 24 hemispheres with arterial vessels branching from the PCA, the origin was exclusively from the PCA in only 5 (20.8%).

In one (1.15%) of the 87 hemispheres, the vessel originated from the medial posterior choroidal artery, and in another hemisphere (1.15%), branches originated from the medial posterior choroidal artery, PCA, MSCA, and LSCA (Table 2).

**Table 2:** Origin of perforators from the PCA.

**Perforators PCA**	**Hemispheres *n =* 24 (100%)**
PCA alone	5 (20.8%)
Medial posterior choroidal artery (MPChoA)	1 (4.16%)
MPChoA + PCA + MSCA + LSCA	1 (4.16%)

*MSCA, medial superior cerebellar artery; LSCA, lateral superior cerebellar artery*.

The following anatomical variations were found in 5 of the 24 hemispheres (Table 3):

- two branches of the PCA in five hemispheres (20.8%);
- an anastomosis among two branches of the PCA and the lateral mesencephalic vein in one hemisphere (4.16%);
- an anastomosis between the PCA and MSCA in one hemisphere (4.16%);
- an “accessory” PCA, which can be considered as a “replaced PCA” supplying the territories of all PCA branches, in 3 (12.5%) hemispheres (a single hemisphere and two hemispheres of a same brain). In the single hemisphere, the replaced PCA was a small-caliber vessel located above the SCA and below the PCA, dorsally and running cranially to the BA. In the one whole brain with a replaced PCA in both hemispheres, we documented eight perforators in the right hemisphere (one directly from the “accessory PCA,” four from a superior branch of the PCA, three from an inferior branch of the PCA), and four perforators directly from the “accessory PCA” in the left hemisphere.

**Table 3:** Anatomical variations in the perforators originating from the PCA.

**Perforators PCA anatomical variations**	**Hemispheres *n =* 5 (100%)**
Two branches	5 (100%)
Anastomosis PCA + lateral mesencephalic	1 (20%)
vein (LMV)	
Anastomosis PCA + MSCA	1 (20%)
“Accessory” PCA	3 (60%)

*MSCA, medial superior cerebellar artery*.

We found no perforators from the collicular artery in any of these hemispheres.

### Origin From Superior Cerebellar Artery

In the 61 hemispheres with vessels branching from the SCA, we found an origin from the MSCA in 56 (91.8%) and the LSCA in 32 (52.46%), in various combinations:

- the origin was exclusively from the MSCA in 32 hemispheres (52.46%) from the LSCA in 15 hemispheres (24.6%);
- vessels branched from both the MSCA and LSCA in 11 hemispheres (18.03%);
- in 3 of the 61 hemispheres (4.92%), we found an anatomical variation represented by a *communicating artery* between the MSCA and LSCA (Table 4).

**Table 4:** Origin of perforators from SCA.

**Perforators SCA (MSCA + LSCA)**	**Hemispheres *n =* 61 (100%)**
MSCA	32 (52.46%)
LSCA	15 (24.6%)
MSCA + LSCA	11 (18.03%)
Anastomosis MSCA + LSCA	3 (4.92%)

*MSCA, medial superior cerebellar artery; LSCA, lateral superior cerebellar artery*.

The analysis of the perforating vessels originating only from the MSCA and LSCA, among the dissected 87 hemispheres, showed this distribution:

- the MSCA presented two and four branches in 15 (17.24%) and two (2.29%) hemispheres, respectively;
- the LSCA presented two branches in eight (9.19%) and four branches in two (2.29%) hemispheres;
- an anastomosis between the MSCA and LSCA was found in three hemispheres (3.44%) (**Figure 3**).

### Origin From Posterior Cerebral Artery and Superior Cerebellar Artery: Combinations

We found an origin from the PCA and SCA (MSCA, LSCA), in various combinations, in 28 of the 87 hemispheres (32.2%) (Table 5).

**Table 5:** Perforators originating from the PCA and SCA in different combinations.

**Perforators PCA + SCA (MSCA + LSCA)**	**Hemispheres *n =* 28 (100%)**
PCA + MSCA	17 (60.71%)
PCA + LSCA	2 (7.14%)
PCA + MSCA + LSCA	9 (32.14%)

*MSCA, medial superior cerebellar artery; LSCA, lateral superior cerebellar artery*.

We documented an origin from both the PCA and MSCA in 17 hemispheres (19.54%), from the PCA and LSCA in 2 (2.29%), and from the PCA, MSCA, and LSCA in 8 hemispheres (9.19%).

### Origin From Basilar Artery

Two (2.29%) of the 87 hemispheres showed perforators originating directly from a small branch of the basilar artery (BA). One (1.14%) had only one perforating vessel, while the other (1.14%) had 4.

### Hemispheres With No Perforating Vessels

We did not find any perforating branches in 7 hemispheres (8.04%).

### Distances in the Lemniscal Trigone Zone

In 32 hemispheres, we were able to measure the superior cerebellar peduncle, lateral mesencephalic sulcus, and inferior brachial colliculus using a digital caliper (Digimatic CD-15B, Mitutoyo, Kawasaki, Japan). We measured the distances between the inferior colliculus and inferior lateral sulcus, the inferior lateral sulcus and superior lateral sulcus, and the inferior colliculus and superior lateral sulcus. The measurements and distances are shown in Table 6.

**Table 6:** Morphometry and measurements of the lemniscal trigone for 32 hemispheres.

**Hemisphere**	**Side**	**Inferior colliculus**	**Inferior lateral sulcus**	**Superior lateral.sulcus**	** $s=\frac{S}{2}=\frac{\left(a+b+c\right)}{2}$ **	**Area, mm^2^**
1	L	7.53	8.11	7.93	11.785	26.654
2	R	11.6	7.52	9.67	14.395	36.152
3	R	8.33	8.27	8.2	12.4	29.587
4	L	7.51	8.4	7.92	11.915	27.148
5	L	7.31	10.3	9.9	13.755	34.362
6	R	9.97	10.98	11	15.975	48.825
7	R	8.27	8.78	9.36	13.205	33.298
8	R	11.48	10.42	8.23	15.065	41.409
9	L	10.91	7.48	8.98	13.685	33.297
10	R	10.95	8.83	10.9	15.34	44.119
11	R	10.64	7.27	9.14	13.525	32.714
12	L	8.2	7.21	8.39	11.9	26.922
13	L	7.96	9	10.9	13.93	35.246
14	L	9.75	10.14	9.81	14.85	42.401
15	R	10.88	8.94	10.66	15.24	43.786
16	R	10.28	7.84	10.7	14.41	38.087
17	L	10.89	11.6	9.55	16.02	48.479
18	L	11.49	9.54	6.36	13.695	30.337
19	R	10.16	9.45	7.85	13.73	35.122
20	R	11.8	8.56	11.69	16.025	46.808
21	R	8.43	12.9	13.25	17.29	52.124
22	R	9.56	9.54	9.27	14.185	38.701
23	L	12.37	7.62	12.55	16.27	45.186
24	L	11.43	11.73	11.3	17.23	57.091
25	L	12.36	13.5	11.67	18.765	67.005
26	L	12.71	12.67	7.7	16.54	46.553
27	R	11.67	10.87	7.3	14.92	38.684
28	R	9.78	12.28	10.19	16.125	48.320
29	R	12.24	11.92	11.38	17.77	60.609
30	R	10.61	8.67	8.68	13.98	36.413
31	L	10.87	13.65	11.5	18.01	60.414
32	L	10.22	8.38	9.44	14.02	37.097
Overall						
Mean (SD)		10.255 (1.576)	9.762 (1.927)	9.730 (1.636)		41.342 (10.328)
Range		12.71–7.31	13.65–7.21	13.25–6.36		67.005–26.654
Variance		2.484	3.713	2.677		106.672

*A, inferior colliculus; B, inferior lateral sulcus; C, superior lateral sulcus; a-b = c, superior cerebral peduncle; B – C = a, lateral mesencephalic sulcus; A – C = b, inferior collicular brachium; s, semi-perimeter of the triangle; S, perimeter of the triangle. Heron's formula states that the area of a triangle whose sides have lengths a, b, and c is $\sqrt{s}\left(s-a\right)\left(s-b\right)\left(s-c\right)]$, where s is the semi-perimeter of the triangle; that is s = (a+b+c)/2*.

## Discussion

In our microanatomical definition, the dorsomedial surface of the mesencephalon is represented superiorly by the superior colliculus and the superior collicular brachium and inferiorly by the inferior colliculus and its brachium. In contrast, the dorsolateral surface is just this small triangular area known as lemniscal trigone or Reil's Trigone (Testut and Latarjet, 1949), delimited posterosuperiorly by the inferior colliculus and its brachium, posteroinferiorly by the superior cerebellar peduncle (brachium conjunctivum), and anteriorly by the lateral mesencephalic sulcus, which is recognized by the lateral mesencephalic vein (LMV), a constant landmark (Ardeshiri et al., 2006, 2007).

We prefer to use the definition “dorsolateral” because it describes our region of interest with the dorsomedial surface. Both parts are located posteriorly (dorsally), but the dorsomedial area is located medially in the three-dimensional topographic planes. Simultaneously, the lemniscal trigone is lateral and separated from the purely lateral surface by the lateral mesencephalic sulcus.

### Perforating Arteries of the Midbrain Have Not Been Named Individually

The vertebrobasilar system provides blood supply to the midbrain through the perforators originating from the BA, SCA, and PCA. The sacrifice of those perforators during the surgical procedure can cause a deficit in part or all the functions carried out by the aforementioned structures. Unfortunately, perforating arteries cannot be identified in the preoperative surgical plan due to the extremely small size, and thus the surgical planning and choice of approach cannot be modified according to the perforators' position and origin.

In the surgical practice, the origin of these perforators from the SCA or PCA usually does not modify the surgical approach, as it is not possible to identify perforators with the current non-invasive diagnostic tools.

The perforating arteries of the brain stem and posterior fossa have been among the most neglected intracranial vascular structures in the anatomical and neurosurgical literature (Matsushima et al., 1983; Rhoton, 2000a). Although essential for surgery and contributing significantly to the operative pathology, they are often referred to only as “perforating arteries” rather than by their proper names (Huang et al., 1968; Graham, 1996).

A crucial artery in this region's anatomy is the pedunculoquadrigeminal artery, also known as collicular artery (Alezais and D'Astros, 1892; Duvernoy, 1978; Yaşargil, 1987). It presents two branches, the main collicular artery and the accessory collicular artery, which, in turn, give rise to four different groups: anteromedial, anterolateral, lateral, posterior. This vessel makes a prominent contribution to the perforating branches group (Yaşargil, 1987).

Special attention is paid to the lateral branches, which, following the main collicular artery, reach the middle segment of the lateral mesencephalic sulcus. Sometimes, some branches that supply to the mesencephalon's dorsolateral surface are present. They give a vast number of small penetrating arteries that anastomose with branches of the posteromedial choroidal artery superiorly and, less frequently, with branches of the SCA inferiorly (Matsushima et al., 1983). However, in our dissections, we did not find any perforators originating directly from the collicular artery or accessory collicular artery.

The veins in this anatomical region have received considerable attention in neuroradiological (Hochstetter, 1938; Scheinker, 1945; Cannon, 1951; Huang and Wolf, 1965; Babin and Megret, 1973; Tamaki et al., 1976; Suzuki et al., 2001; Ardeshiri et al., 2007; Wackenheim and Braun, 2013) and anatomical studies (Stephens and Stilwell, 1969; Duvernoy, 1974, 1978; Lang et al., 1981; Matsushima et al., 1983; Ono et al., 1984; Graham, 1996; Tomasi et al., 2020). However, not much data about the course, origin, anatomical variations, and frequency of the dorsal perforating arteries could be found in the literature, especially concerning the lemniscal trigone.

The NIH Traumatic Coma Data Bank (concerning many patients with severe head injuries) stated, in 1990, that intracranial hypertension and death are strongly correlated with compression or obliteration of the mesencephalic cisterns. These findings corroborate the critical role of midbrain swelling or displacement (Eisenberg et al., 1990; Graham, 1996).

Further studies indicate that obstructions in the deep venous system can also lead to *diffuse axonal degeneration* (Graham, 1996). This degeneration of the white matter is localized and concerns the periventricular matter drained by the deep venous system, sparing the subcortical fibers (Jellinger and Seitelberger, 1970). This phenomenon and the involvement of cortical areas drained mostly by the deep venous system indicate that obstruction of veins of the deep system can cause white matter degeneration (Andeweg, 1999).

Concerning the perforating arteries in the dorsal perforation areas, Yaşargil describes two zones, each divided into three groups (Yaşargil, 1987) (Table 7):

1. Dorsal thalamic perforation zone, which includes:
  - medial posterior choroidal group;
  - lateral posterior choroidal group;
  - cingulothalamic group;
2. Dorsal midbrain perforation zone, including:
  - pericollicular groups;
  - circumgeniculate groups;
  - lemniscal trigone group.

**Table 7:** Yasargil description of perforating arteries in the dorsal perforation zones.

**Dorsal thalamic perforation zone Dorsal midbrain perforation zone**	**Dorsal thalamic perforation zone** **Dorsal midbrain perforation zone**
Medial posterior choroidal group pericollicular groups	Medial posterior choroidal group pericollicular groups
Lateral posterior choroidal group circumgeniculate groups	Lateral posterior choroidal group circumgeniculate groups
Cingulothalamic group leminiscal trigone group	Cingulothalamic group leminiscal trigone group

As described in detail by Rhoton, lesions of the posterior incisura include pineal tumors, meningiomas originating at the falcotentorial junction or at the edge of the tentorium, as well as from the tela choroidea of the velum interpositum and atrium (Ono et al., 1984; Rhoton, 2000b). Also, deep-sited gliomas can be found at the level of the splenium, pulvinar, and quadrigeminal plate. Regarding vascular pathologies, aneurysms of the vein of Galen and arteriovenous malformations can require surgical or endovascular treatment. The microsurgical resection of those lesions must spare the perforator arteries of the lemniscal trigone in order to achieve a safer resection.

### Operative Approaches and Safe Entry Zones

The possible surgical approaches are interhemispheric transtentorial, interhemispheric transcallosal, transventricular, subtemporal transtentorial, retrosigmoid, anterior petrosectomy, retrolabyrinthine, amd infratentorial supracerebellar and its extreme-lateral variation.

Lesions may be reached cranially from the tentorium using an occipital transtentorial approach, a posterior transventricular approach, or a posterior interhemispheric transcallosal approach. Caudally from the tentorium, possible approaches are the infratentorial supracerebellar and the occipital transtentorial, in some cases associated with the incision of the tentorium laterally to the straight sinus. These approaches are commonly used to approach midline tumors like pineal lesions and for lesions located below the vein of Galen and its tributaries (Stein, 1979). PCA or SCA tentorial branches entering the dura laterally to the straight sinus must be considered.

For lesions sited at the level of the tentorial edge, the occipital transtentorial approach is better suited, particularly if the vein of Galen is located below the lesion. Furthermore, this approach provides a favorable corridor for lesions involving the ipsilateral half of the cerebellomesencephalic fissure and the ambient cistern posteriorly, even if they can be below the vein of Galen (Poppen, 1966; Yasargil et al., 1976).

In the case of lesions originating from the splenium and above the vein of Galen, developing into the posterior incisura, the posterior transcallosal approach with division of the splenium can be an appropriate choice.

For anterolateral midbrain lesions, a subtemporal approach could be the possible choice, whereas, for posterolateral or posterior lesions, the approach of choice could be the supracerebellar infratentorial, paramedian, median, or its extreme-lateral variation.

In case of lesions involving the anterolateral surface of the pons, the subtemporal, transtentorial, retrolabyrinthine, retrosigmoid approaches, or the anterior petrosectomy are all viable options.

**Figure 1:**
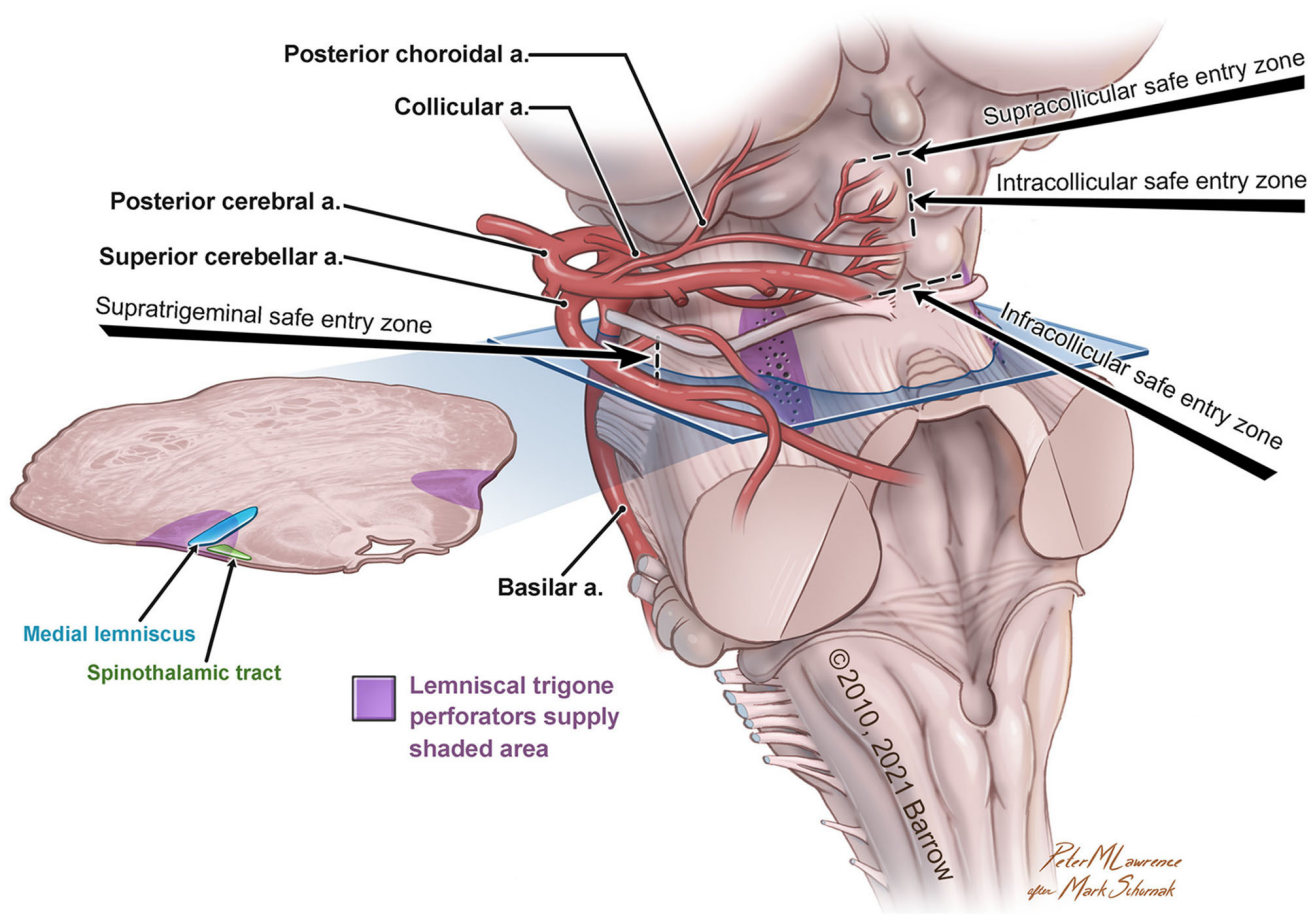
Anatomical illustration of the lemniscal trigone zone. a., artery. Used with permission from Barrow Neurological Institute, Phoenix, Arizona.

The variability in the choice of approach is correlated not only to the lesion but also to the accessible safe entry zones in the region of interest (Figure 1).

For approaching the anterolateral midbrain area, we propose four different possible safe entry zones.

The *supratrigeminal entry zone* could be accessed by subtemporal transtentorial, retrosigmoid, retrolabyrinthine approach, or anterior petrosectomy. This corridor is an advantageous entry zone to reach lesions placed anteriorly in the pons, just above the trigeminal root entry zone to the middle cerebellar peduncle (Hebb and Spetzler, 2010; Cavalcanti et al., 2016). Due to the middle cerebellar peduncle's posterolateral location and the relative thickness of the pontine transverse fibers, the pons could be reached through a meticulous dissection along these fibers medially, anteromedially, or posteriorly to the corticospinal tract (Poppen, 1966).

The other safe entry zones playing an important role in the lemniscal trigone region are the *supra-, intra-, and infra-collicular zones*, which could be reached with the supracerebellar infratentorial and the extreme-lateral supracerebellar approaches.

First described by Kyoshima et al. (1993) and later, with a very detailed anatomo-morphological study, by Strauss et al. (1997), the supracollicular and the infracollicular safe entry zones are represented by the small areas above the superior colliculi and below the inferior colliculi, respectively.

The intracollicular safe entry zone was firstly described by Bricolo and Turazzi (Bricolo et al., 1991; Bricolo and Turazzi, 1995) and later supported by other studies (Cantore et al., 1999; Kumar and Singhi, 2004; Ramina et al., 2011; Yagmurlu et al., 2014; Cavalcanti et al., 2016; Kalani et al., 2016; Matsushima et al., 2016), and it represents a small area between the two zones mentioned above. This entry zone is considered a safe corridor because of its sparseness of fibers.

A crucial artery as parent vessel for the perforators of the lemniscal trigone is the pedunculoquadrigeminal artery, also known as collicular artery (Alezais and D'Astros, 1892; Duvernoy, 1978; Yaşargil, 1987). The collicular artery has two branches, the main colicular artery and the accessory collicular artery, which, in turn, give rise to four different groups: anteromedial, anterolateral, lateral, posterior. This vessel heavily contributes to perforating vessels (Yaşargil, 1987).

### Perforators Origin Not Only From the SCA

**Figure 2:**
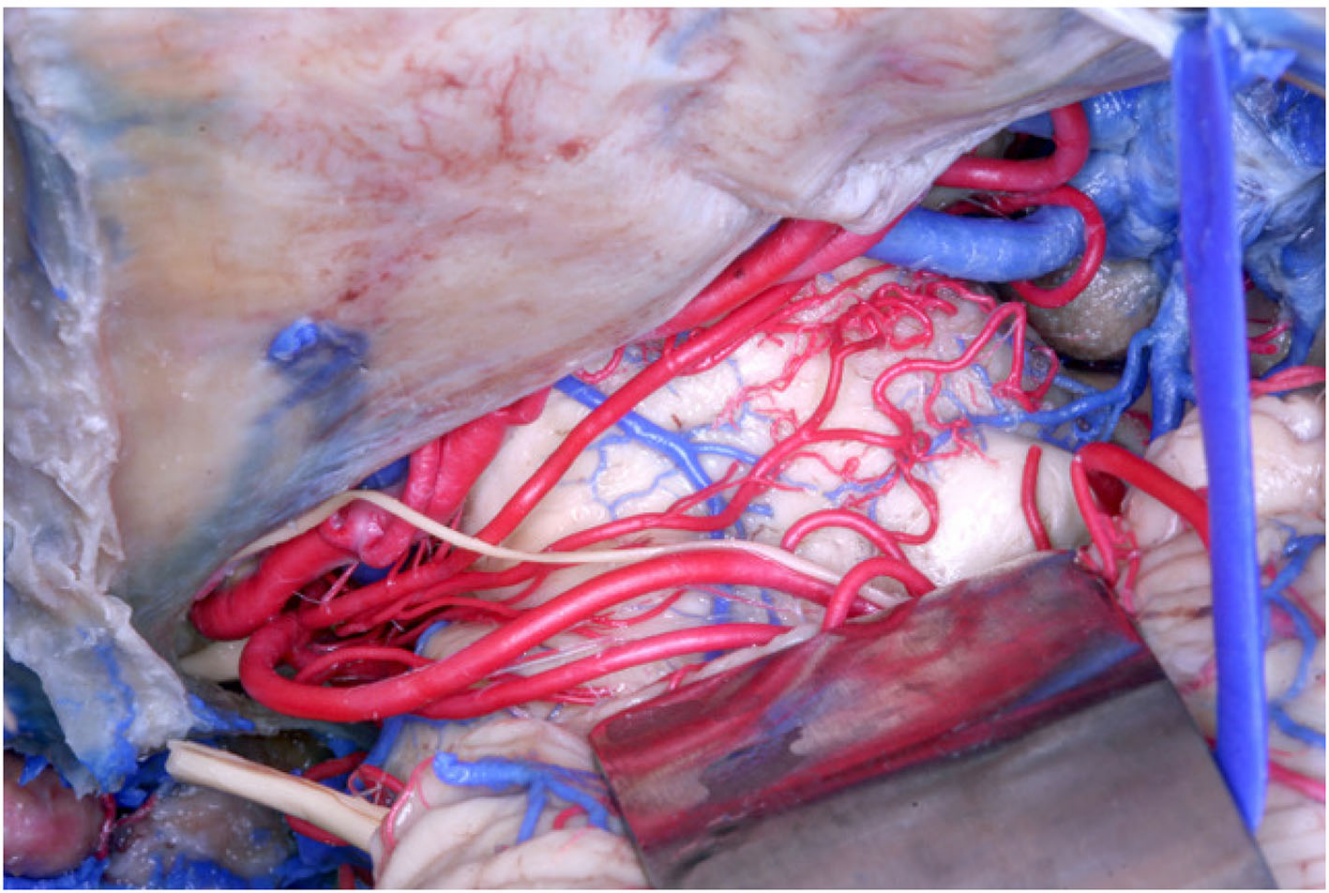
Anatomical picture of the microsurgical dissection of the left lemniscal trigone zone. We can see the tentorium superiorly, which covers a part of the PCA (conventionally injected in red). Below the PCA we see the SCA (conventionally injected in red), which divides into two branches, the MSCA and the LSCA. The image shows the perforators from the SCA. The LMV (conventionally injected in blue) is very well-visualized, as well the trochlear nerve (IV), which crosses the PCA and runs below the MSCA following the LSCA.

In our study, following Duvernoy (1978) and Yaşargil (1987), we documented an origin from the SCA in 61 (70.11%) of the 87 hemispheres, but we further described other parent vessels from which perforators originated. Specifically, among the 61 hemispheres, an origin from the MSCA was found in 56 (91.8%) hemispheres, and from the LSCA in 32 hemispheres (52.46%), whereas an origin exclusively from the MSCA and from the LSCA was found in 32 (52.46%) and 15 (24.6%) hemispheres, respectively (Figure 2). In 11 hemispheres (18.03%), the origin was from both the MSCA and LSCA.

**Figure 3:**
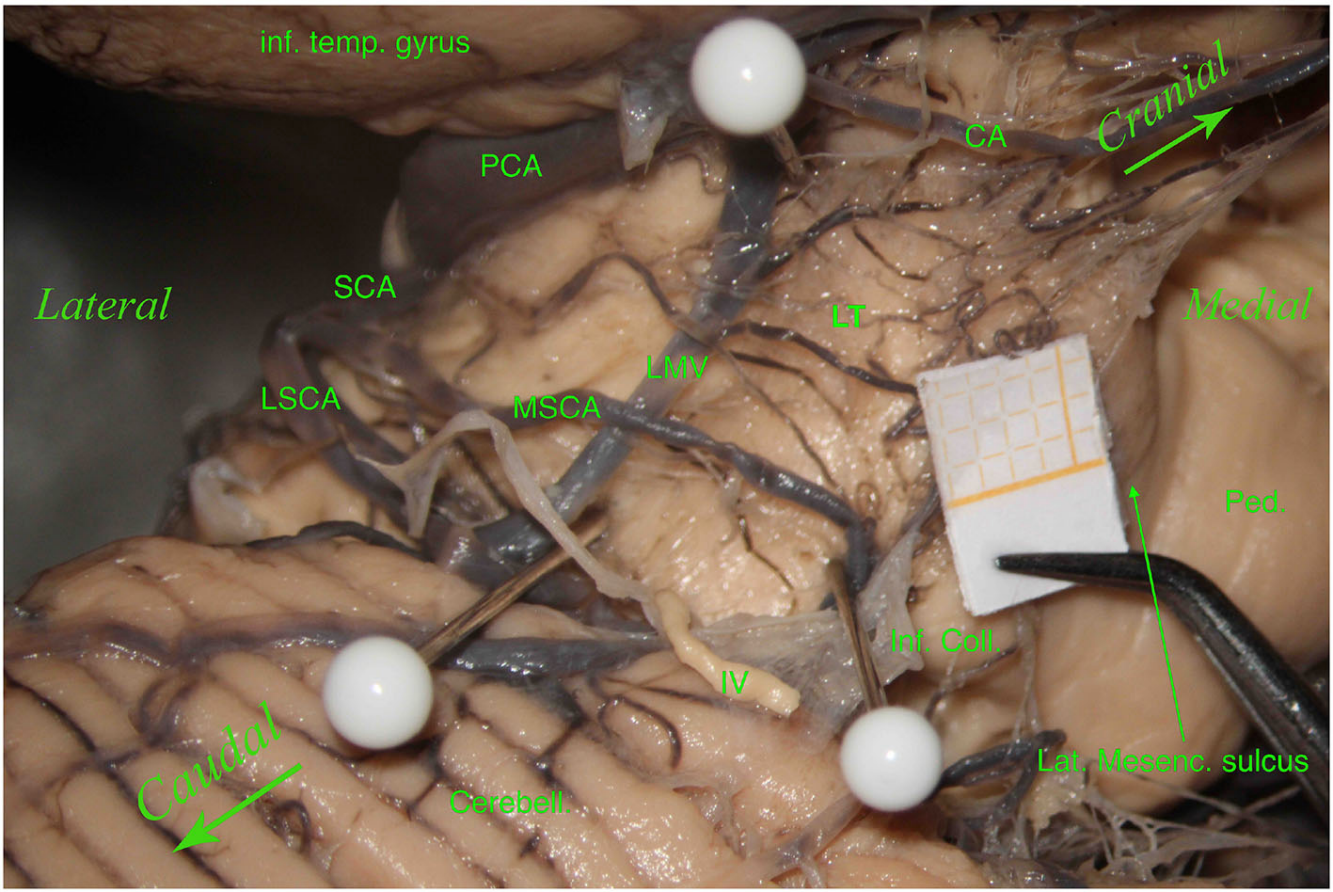
Left hemisphere. From top to bottom and from left to right, we see the inferior temporal gyrus (inf. temp. gyrus), the PCA, the SCA, the lateral mesencephalic (Lat. Mesenc.) vein (LMV), the remnants of a damaged trochlear nerve (IV). The SCA divides into two branches, the MSCA and the LSCA. The LSCA has two other branches, superior and inferior. For illustrative purposes, we call these branches medial and lateral (mLSCA and lLSCA). The branches of the LSCA “hug” the LMV: the mLSCA runs behind the LMV, and the lLSCA moves above the LMV. On the upper left, the first white pin is placed at the beginning of the LMV and under the PCA; the second white pin, on the lower left, is placed at the end of the LMV and above the inferior branch of the LSCA. These two pins highlight the lemniscal trigone's anterior border. On the right side, the third white pin is just below the MSCA, showing the inferodorsal part of the lemniscal trigone. Among residual layers of arachnoid, we can appreciate many perforators, mostly—by Duvernoy—coming from the SCA and supplying manly the superomedial surface of the lemniscal trigone. Inf. Coll., inferior colliculus.

Interestingly, we also found an anatomical variation with a “bridging artery,” an anastomosis between the MSCA and LSCA, in 3 of the 61 hemispheres (4.92%) (Figure 3).

**Figure 4:**
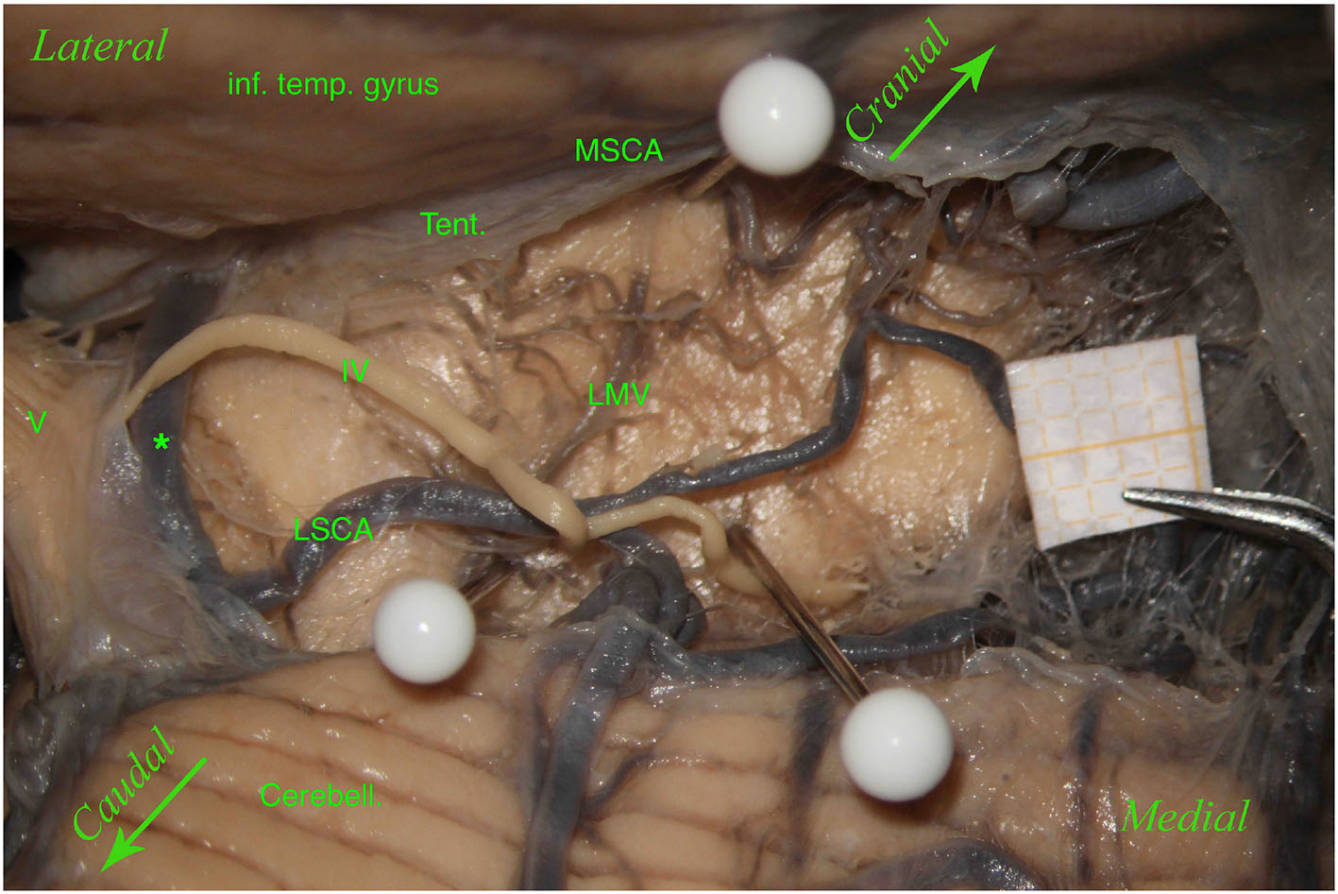
Left hemisphere. From top to bottom: the inferior temporal gyrus (inf. temp. gyrus), remnants of the tentorium covering the MSCA, the cerebellum (Cerebell.). The first white pin is placed at the root of the LMV, whereas the second white pin is located where the LMV ends. The two pins highlight the course of the LMV. In the middle, between the inferior temporal gyrus and the cerebellum, from left to right, we can see the trigeminal nerve (V), an anastomosis between the MSCA and the LSCA (*), and a partially removed trochlear nerve (IV). The trochlear nerve runs above the anastomosis and the LSCA and between the two branches of the LSCA—superior and inferior (third white pin). In this specimen, the perforators come mostly from the MSCA and distribute in the lemniscal trigone's superior part; only a small group comes from the LSCA.

Unlike Duvernoy (1978) and Yaşargil (1987), we found perforating vessels originating from the posterior cerebral artery (P2 segment) in 24 hemispheres (27.58%) and from the basilar artery in 2 (2.29%) of the 87 hemispheres (Figure 4).

**Figure 5:**
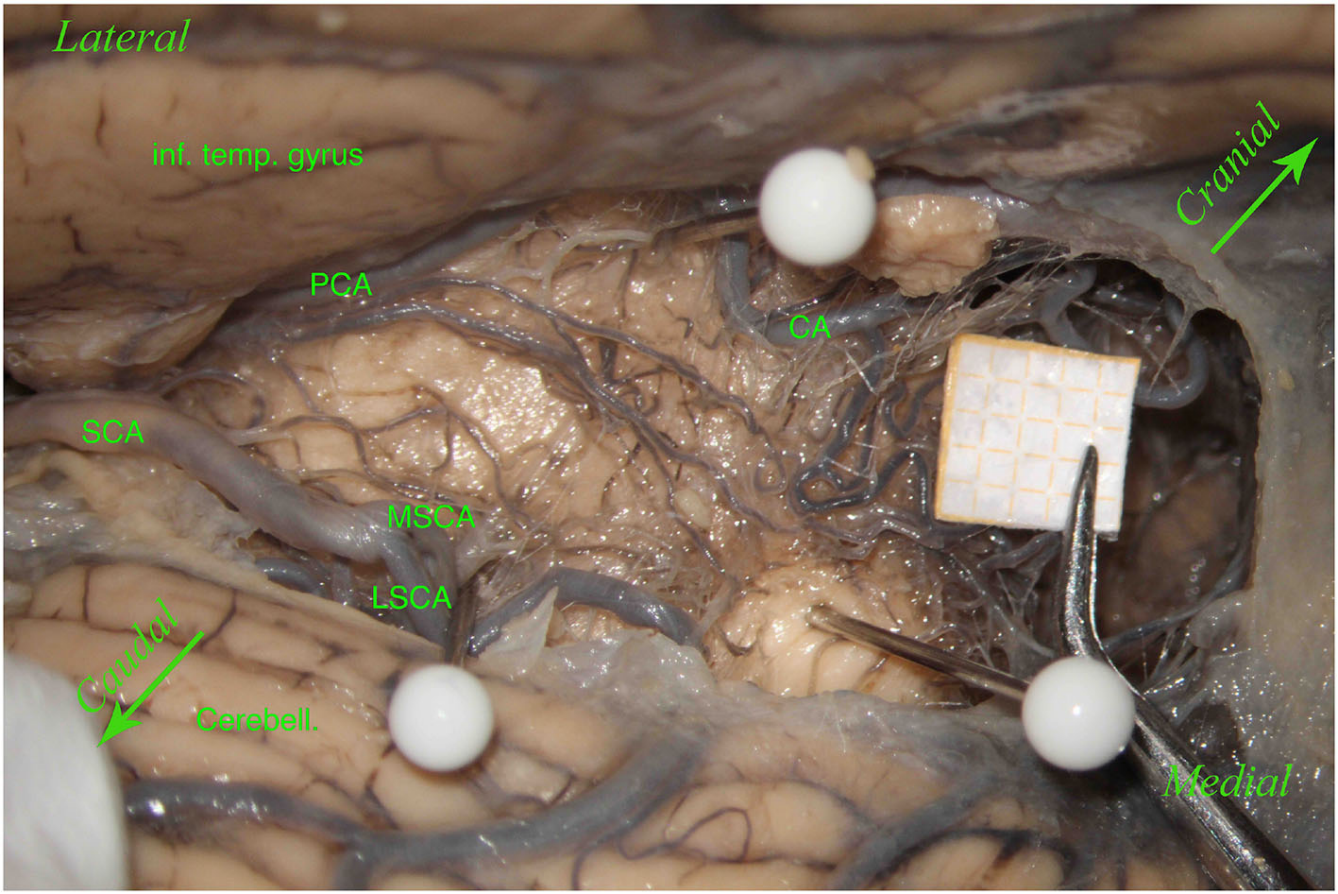
Left hemisphere. The inferior temporal gyrus (inf. temp. gyrus) is seen from the top and the left, covering the PCA, which gives many perforators. Below there is the SCA with a smaller MSCA and a bigger LSCA. The upper white pin, below the inferior temporal gyrus, between the PCA and the CA, shows the origin of the LMV; the lower white pin, above the cerebellum (Cerebell.), the MSCA, and the LSCA, shows the end of the LMV. These two pins, showing the LMV, represent the lemniscal trigone's anterior border. In this specimen, the LMV is very small. The third white pin represents the inferior dorsal part of the lemniscal trigone. In this hemisphere, unlike the other left hemisphere described in Figure 1 and differently from Duvernoy's illustrations, perforators come mostly from the PCA and a minority from the MSCA, and only a few from the LSCA. Two big perforating branches from the PCA run above the LMV to reach the middle dorsal part of the lemniscal trigone, together with the other perforators coming mostly from the MSCA. This anatomical aspect could be of clinical significance because, in this case, damage in one of these two groups of perforators does not necessarily give a clinical manifestation related to the auditory pathway.

Specifically, although in other studies the lemniscal trigone's perforating group represents the entry point of branches from the SCA and the collicular artery (when present) (Matsushima et al., 1983; Rhoton, 2000a), we found an origin exclusively from the PCA in 5 hemispheres (20.8%) (Figure 5).

Furthermore, in one (1.14%) of the 87 hemispheres, we found an origin from the medial posterior choroidal artery and, in another hemisphere (1.14%), branches emanating from the medial posterior choroidal artery, PCA, MSCA, and LSCA.

**Figure 6:**
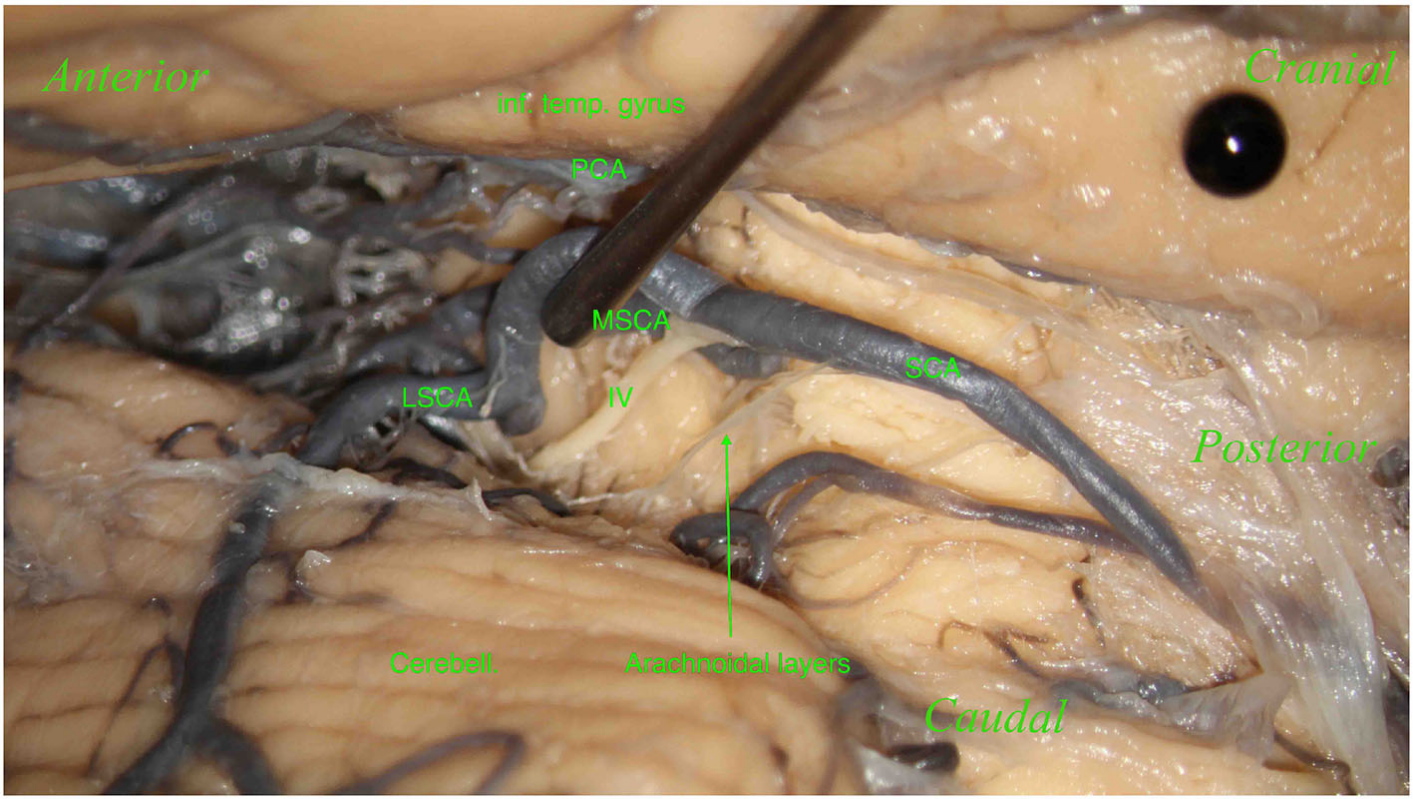
Right hemisphere. From top to bottom: the inferior temporal gyrus (inf. temp. gyrus; black pin), covering the PCA and the cerebellum (Cerebell.). From right to left: the SCA and its two branches: the MSCA and the LSCA. The image shows a part of the trochlear nerve (IV). Below, the SCA, dividing into its two branches, the MSCA and LSCA. No perforators in this hemisphere were found.

No perforating branches were found in 7 hemispheres (8.04%) (Figure 6).

The knowledge of the lemniscal trigone's perforating arteries and their origin, together with the various anatomical variations and the impossibility to visualize these small vessels in the preoperative assessment (Alezais and D'Astros, 1892; Testut and Latarjet, 1949; Duvernoy, 1978; Ardeshiri et al., 2006, 2007), should be taken into account when performing surgery in this challenging area (Bricolo et al., 1991; Kyoshima et al., 1993; Bricolo and Turazzi, 1995; Ramina et al., 2011; Yagmurlu et al., 2014; Cavalcanti et al., 2016; Kalani et al., 2016; Matsushima et al., 2016): sparing these perforators could help in reducing surgical morbidity and improving clinical outcome.

### Study Limitations

Our study's limitations are related to the fact that this a purely anatomical study, without any clinical-surgical correlation. The clinical impact related to damage to perforating arteries is hypothetical and strictly associated with the auditory tracts underneath the trigone and relevant nuclei and fibers surrounding the area.

The lemniscal trigone is a small area, sometimes challenging to reach without damaging the extremely small perforators, which cannot be visualized with the current preoperative tools. Thus, the surgical approach cannot be planned based on preoperative imaging. The surgeon should deal with the perforators documented intraoperatively.

**Figure 7:**
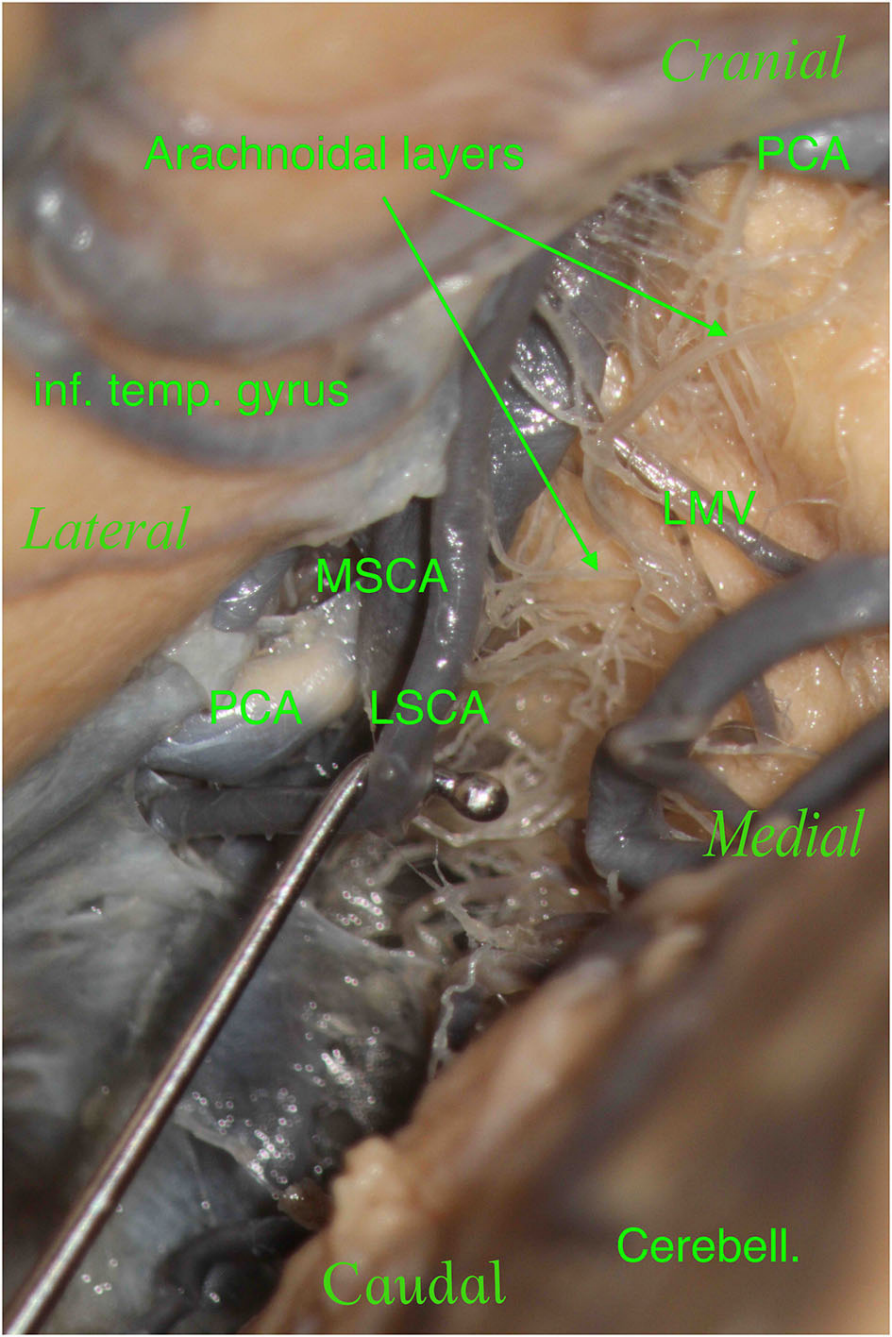
The right hemisphere, oblique view. On the top left, we see the inferior temporal gyrus (inf. temp. gyrus), which partially covers the PCA. On the bottom right, we see the cerebellum (Cerebell.). We see a tiny space where our main vessels are located in this specimen: the PCA and SCA with their two branches, the MSCA and LSCA. We moved and lifted the SCA to see the multitude of arachnoidal layers present in this specimen: this is a crucial point.

**Figure 8:**
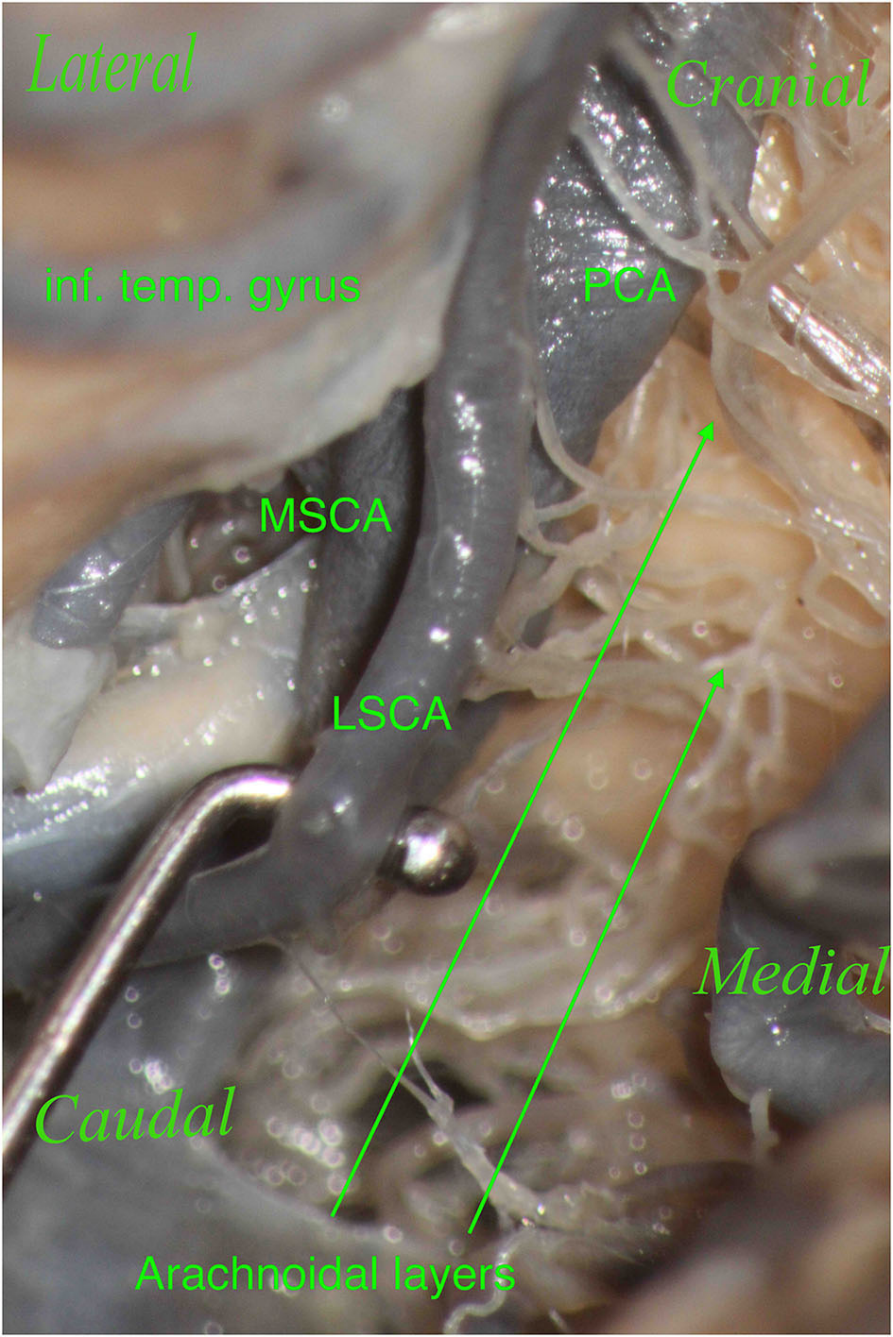
The right hemisphere, oblique view. Particular: magnification of the arachnoidal layers illustrated in Figure 7. During dissection, it is essential to clean this region properly to show the perforators, which should not be confused with the arachnoidal layers and vice versa.

Furthermore, during dissection, it is essential to clean this region properly in order to show the perforators, which should not be confused with the arachnoidal layers and vice versa (Figures 7, 8). The differentiantion can be made by proper exposure of the possible parent vessel. The present study offers the opportunity to be aware of the possible contributors to the perforators of the lemniscal trigone, by frequency, thus suggesting the parent vessel to be exposed with the greater chance to originate the perforators.

Given that knowing the anatomy is the prerequisite for a safer surgery, we encourage new microanatomical studies in this area to better understand the dorsolateral perforating arteries in the lemniscal trigone zone.

## Conclusions

The novelty of this study is represented by the first detailed description of the perforating vessels of the lemniscal trigone, in relation to their parent vessels.

In a more detailed analysis, although in other studies the lemniscal trigone's perforating group represents the entry point of branches from the SCA and the collicular artery (when present), we also found an origin exclusively from the PCA in 5 (20.8%) of our 87 hemispheres.

Furthermore, as shown in Table 5, PCA + MSCA account for more than 60% of the perforators' origin. Thus, special attention should be paid during surgery to spare those vessels and the associated perforators.

A thorough understanding of the perforating arteries' microanatomy in the lemniscal trigone is crucial in brain stem surgery to avoid brain damage, which may be due to hemorrhage or ischemia, following a restriction or a failure in the blood supply to the brain stem. Knowledge of the origin, course, variations, and anastomotic system of these vessels could lead to a better surgical outcome; therefore, special attention should be paid to the perforating arteries during surgical procedures in this challenging region of the brain.

## Data Availability Statement

The original contributions presented in the study are included in the article/supplementary material, further inquiries can be directed to the corresponding author/s.

## Author Contributions

ST: idea, microsurgical anatomical dissections, and writing and revision. PW and ML: critical revision and supervision. KY and RR-R: independent revision. BC: bibliography. PG and JR: pictures. GR and GS: tables. GU: writing. All authors contributed to the article and approved the submitted version.

## Conflict of Interest

The authors declare that the research was conducted in the absence of any commercial or financial relationships that could be construed as a potential conflict of interest.

## Publisher's Note

All claims expressed in this article are solely those of the authors and do not necessarily represent those of their affiliated organizations, or those of the publisher, the editors and the reviewers. Any product that may be evaluated in this article, or claim that may be made by its manufacturer, is not guaranteed or endorsed by the publisher.
