# Corrigendum: Perforating Arteries of the Lemniscal Trigone: A Microsurgical Neuroanatomic Description

**DOI:** 10.3389/fnana.2021.835799

**Published:** 2022-01-05

**Authors:** Santino Ottavio Tomasi, Giuseppe Emmanuele Umana, Gianluca Scalia, Roberto Luis Rubio-Rodriguez, Giuseppe Raudino, Julian Rechberger, Philipp Geiger, Bipin Chaurasia, Kaan Yagmurlu, Michael T. Lawton, Peter A. Winkler

**Affiliations:** ^1^Department of Neurological Surgery - Christian Doppler Klinik, Salzburg, Austria; ^2^Department of Neurosurgery, Paracelsus Medical University Salzburg, Salzburg, Austria; ^3^Laboratory for Microsurgical Neuroanatomy - Christian Doppler Klinik, Salzburg, Austria; ^4^Department of Neurosurgery, Cannizzaro Hospital, Trauma Center, Gamma Knife Center, Catania, Italy; ^5^Neurosurgery Unit, Highly Specialized Hospital and of National Importance “Garibaldi”, Catania, Italy; ^6^Skull Base and Cerebrovascular Laboratory, University of California, San Francisco, San Francisco, CA, United States; ^7^Department of Neurological Surgery, University of California, San Francisco, San Francisco, CA, United States; ^8^Department of Otolaryngology - Head and Neck Surgery, University of California, San Francisco, San Francisco, CA, United States; ^9^Department of Neurosurgery - Humanitas, Istituto Clinico Catanese, Catania, Italy; ^10^Department of Neurosurgery, Neurosurgery Clinic, Birgunj, Nepal; ^11^Department of Neurosurgery, University of Virginia, Charlottesville, VA, United States; ^12^Department of Neurosurgery, Barrow Neurological Institute, St. Joseph's Hospital and Medical Center, Phoenix, AZ, United States

**Keywords:** lemniscal trigone, dorsolateral midbrain perforating zone, microsurgical anatomy, arterial capillary network, perforating arteries, anatomical variability

In the original article, there was a mistake in Figure 1 as published. The word trigone (in purple type) was misspelled (“trigon”). The corrected Figure 1 appears below.

**Figure 1:**
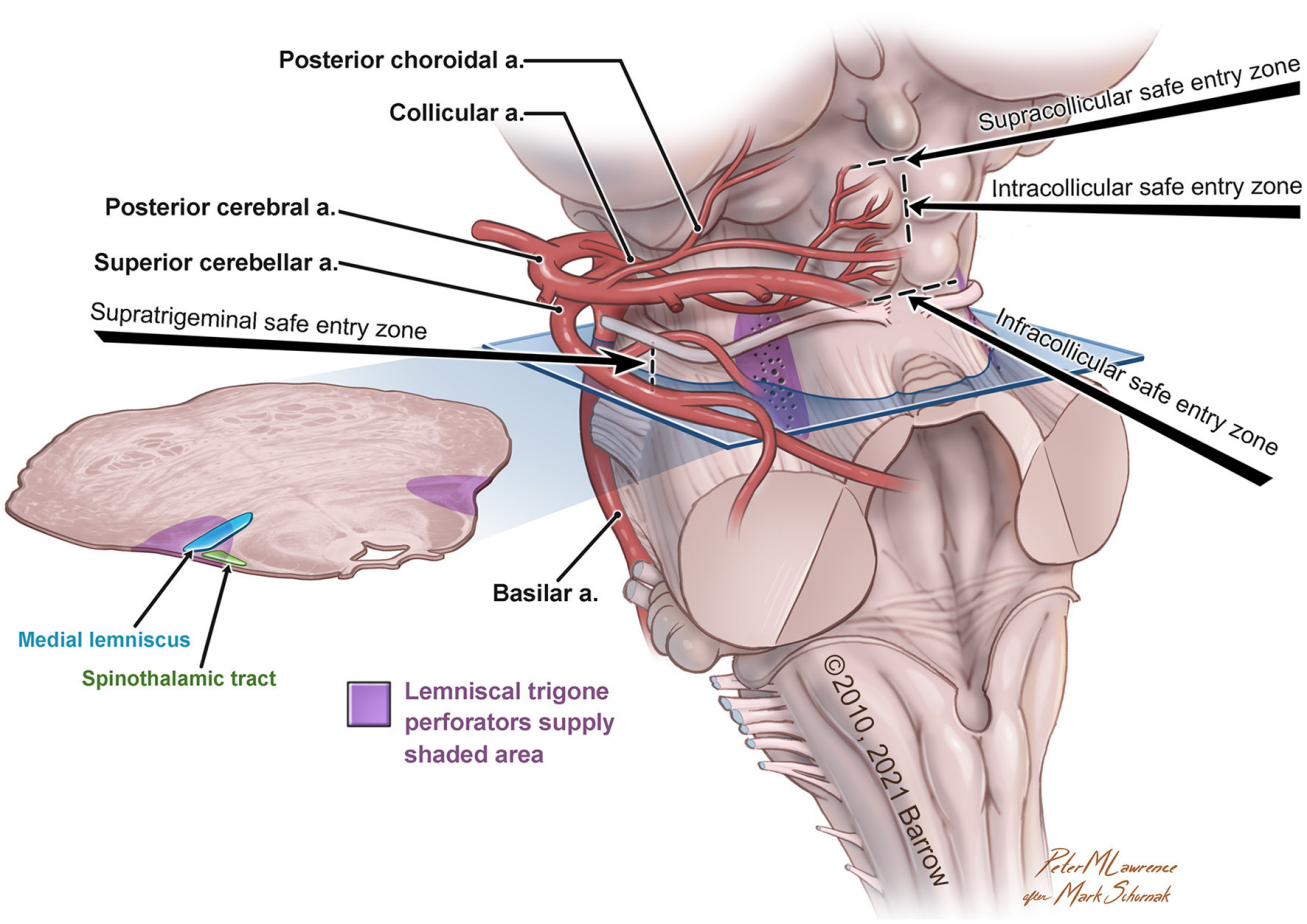
Anatomical illustration of the lemniscal trigone zone. a., artery. Used with permission from Barrow Neurological Institute, Phoenix, Arizona.

The authors apologize for this error and state that this does not change the scientific conclusions of the article in any way. The original article has been updated.

## Publisher's Note

All claims expressed in this article are solely those of the authors and do not necessarily represent those of their affiliated organizations, or those of the publisher, the editors and the reviewers. Any product that may be evaluated in this article, or claim that may be made by its manufacturer, is not guaranteed or endorsed by the publisher.

